# Epidemiology, treatment patterns and healthcare utilizations in multiple sclerosis in Taiwan

**DOI:** 10.1038/s41598-021-86347-3

**Published:** 2021-04-08

**Authors:** Chia-Yun Hsu, Long-Sun Ro, Li-Ju Chen, Chun-Wei Chang, Kuo-Hsuan Chang, I-Hsuan Wu, Amy Lin, Fei-Yuan Hsiao

**Affiliations:** 1 Health Data Research Center, National Taiwan University, Taipei, Taiwan; 2 Department of Neurology, Chang Gung Memorial Hospital, Taipei, Taiwan; 3 Merck Ltd, Taipei, Taiwan; 4 Graduate Institute of Clinical Pharmacy, College of Medicine, National Taiwan University, Room 220, 33, Linsen S. Rd, Taipei, 10050 Taiwan; 5 School of Pharmacy, National Taiwan University, Taipei, Taiwan; 6 Department of Pharmacy, National Taiwan University Hospital, Taipei, Taiwan

**Keywords:** Neurological disorders, Neurological disorders, Inflammation

## Abstract

“Real-world” data on the nationwide epidemiology and treatment patterns of multiple sclerosis (MS) is very scarce in Asia. This study is aim to evaluate the 10-years trends in epidemiology and treatment patterns of MS with Taiwan’s National Health Insurance Database (NHIRD). Patients aged 20 years or older and were newly diagnosed with MS between 2007 and 2016 were identified. The crude incidences of MS were presented annually and stratified by sex and age. Baseline characteristics and treatment patterns, particularly disease-modifying drugs (DMDs), were also analyzed. This study included 555 MS patients (mean age was 36.9 and 74.4% were female). The crude incidence rate of MS decreased slightly from 0.43 per 100,000 persons in 2007 to 0.24 per 100,000 persons in 2015. The female to male ratios remained mainly between 2 to 3. Approximately 80% of MS patients received initial DMDs, with interferon β-1a as the dominant one. Furthermore, 37.5% of MS patients received subsequent DMDs, with fingolimod being the most frequently used. The median times from diagnosis to initial and to subsequent DMDs were 77 and 1239 days, respectively. This nationwide study provides up-to-date and sophisticated estimates of MS epidemiology and treatment pattern in “real-world” setting in Taiwan.

## Introduction

Multiple sclerosis (MS) is a chronic progressive, demyelinating, inflammatory disease of the central nervous system (CNS) and is one of the most frequently seen nontraumatic neurologically disabling disorders that patients have to cope with during their primary working time^1^. MS places heavy disease and economic burden not only on patients and their family but also on society^1–4^.

Ongoing surveillance of the incidence and prevalence of MS is thus critical for health policy and research. Approximately 2.3 million people worldwide were estimated to be affected by MS in 2013, with a global median prevalence of 33 per 100,000 persons. Substantial variations were noticed in both the prevalence and the incidence of MS worldwide, with the prevalence varying from 2.2 per 100,000 persons in East Asia to 140 per 100,000 persons in North America, and the incidence ranging from as low as 0.07 per 100,000 persons in Guatemala to as high as 13.75 per 100,000 persons in San Marino^5^.

These geographical variations in the incidence and prevalence of MS have resulted in an imbalance of “real-world data” of MS to support clinical practice and health policy among different countries. Due to the high incidence and prevalence of MS in North America and Europe, studies regarding the epidemiology and disease burden on MS have been thoroughly conducted and updated during the past few decades^1–10^. In contrast, studies focused on MS in Asia were very limited^11,12^. Nonetheless, some previous studies conducted in Asia still suggested an increasing trend in the prevalence and incidence of MS over the past couple of years^13–19^. The underlying cause of the increase remains obscure and is thought to be beyond the mere evolution of diagnostic criteria^13^. These findings highlight the need for more studies on MS, particularly in Asia.

Another unaddressed issue in Asia is the misclassification between MS and neuromyelitis optica spectrum disorder (NMOSD) due to the similarity in their clinical presentations^20^. This issue is particularly crucial in Asia, as the ratios of NMOSD to MS were higher in Asia than in North America or Europe^21^. To our knowledge, most of the epidemiological studies on MS carried out in Asia did not preclude or take patients with NMOSD diagnosis into consideration^14–17,19^, which may overestimate the incidence of MS.

In addition to epidemiology, an understanding of current management of MS, such as treatment patterns of disease-modifying therapies (DMTs), would be essential to clinicians and health policy makers. For example, DMTs have provided clinical benefits, including reducing the number and severity of relapses and improving the quality of life in patients with MS. However, real-world evidence has suggested that patients having poor adherence to DMTs were associated with worse clinical outcomes^22–25^. Unlike Western countries^26–29^, studies investigating the current management of MS in Asia are very limited.

To fill the current knowledge gap and provide insights into the epidemiology and current management of MS, we used Taiwan’s National Health Insurance Database (NHIRD) to conduct a nationwide study with the following objectives: (a) to evaluate the epidemiology of MS; (b) to examine treatment patterns of DMTs; and (c) to estimate healthcare utilization among incidence cases of MS in Taiwan.

## Methods

### Data source

We utilized data from the NHIRD from January 1, 2006 to December 31, 2016 as our data source. The NHIRD is a nationwide database containing claims data of all beneficiaries enrolled in the National Health Insurance (NHI) program in Taiwan, provided by the Health and Welfare Data Science Center of the Ministry of Health and Welfare, Taiwan. The NHI program was launched in 1995 and covers over 99% of the population in Taiwan (23.6 million in 2018). The NHIRD comprises detailed information on demographics and healthcare utilization, including outpatient visits, hospital admissions, and prescription medications^30,31^. As MS in Taiwan is categorized as one of the Catastrophic Illnesses, we further linked the NHIRD to the Registry for Catastrophic Illness to further confirm the MS patients identified in our study.

### Ethical statement

The protocol of this study was approved by the Research Ethics Committee of National Taiwan University Hospital (registration number, 201712110 W). We confirm that all research was performed in accordance with relevant guidelines/regulations. The identification numbers of the beneficiaries were encrypted to ensure their confidentiality and thus the Research Ethics Committee of National Taiwan University Hospital has waived the requirement of informed consent for the study.

### Study population

**Figure 1:**
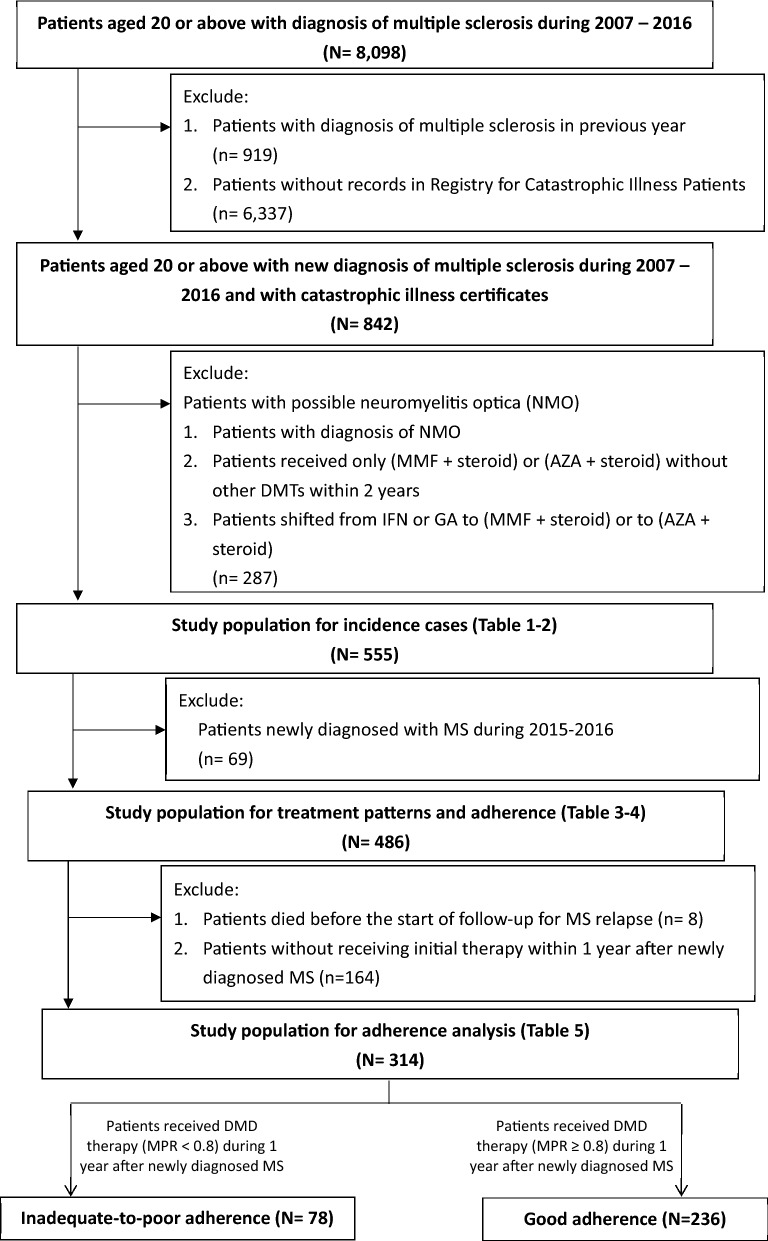
Flow chart describing the enrollment of the study population.

Patients aged 20 years or older with MS diagnosis upon ambulatory care visits and inpatient admissions were identified using the ICD-9-CM code (340) and ICD-10-CM code (G35) between 2007 and 2016. The first MS diagnosis date during 2007–2016 was defined as the cohort entry date. Patients who had any MS diagnosis before the cohort entry date were excluded to identify incidence cases of MS. Those who did not have records in the Registry for Catastrophic Illness were further excluded. We further excluded patients who had a diagnosis of NMO (ICD-9-CM code 341, ICD-10-CM code G36), those who received only mycophenolate mofetil (MMF), steroid or azathioprine (AZA) or steroid but without other DMTs within two years following the cohort entry date, and those who shifted from interferon beta (IFN β) or glatiramer acetate (GA) to MMF plus steroid or AZA plus steroid to ensure that our study population consisted of MS patients but not NMO patients (Fig. 1).

### Incident cases and their characteristics

Incident cases between 2007 and 2016 were reported annually. Baseline characteristics were collected within one year prior to the date of newly diagnosed MS. Information on pregnancy and comorbidities, including psychotic symptoms (depression and anxiety), cardiovascular disease (hypertension, ischemic heart disease and heart failure, cardiac dysrhythmia), endocrine disease (dyslipidemia and diabetes mellitus), obstructive lung disease (COPD and asthma), autoimmune disease, malignancy, traumatic brain injury and prior health behaviors such as the use of oral contraception, were gathered.

### Treatment patterns

To guarantee that each incidence case had at least 2 years of follow-up, in the analyses investigating treatment patterns and adherence, we excluded incidence cases during 2015–2016. DMTs, including IFN β-1a, IFN β-1b, GA, fingolimod, natalizumab, and other possible agents that might be prescribed in MS patients, including mitoxantrone, rituximab, AZA, and MMF, were included in this study.

The first agent initiated immediately after the cohort entry date was defined as the initial therapy. If a patient discontinued the initial therapy, we further identified his or her subsequent therapy. The time from the diagnosis of MS to initial therapy and the time from initial therapy to subsequent therapy were also analyzed.

The association between adherence to initial therapy (within 1 year after the diagnosis of MS) and the risk of MS relapse was further examined. The medication possession ratio (MPR) was used to measure adherence to DMTs, which is defined as the ratio of days of medication supply to a given time interval^32^. We reported adherence at 12 and 24 months following the initial and subsequent DMTs. Adherence to DMTs was categorized as the good (MPR ≥ 0.8), inadequate (0.8 > MPR ≥ 0.4) and poor (MPR < 0.4) adherence groups. All patients were followed up from 12 months after initial therapy to the occurrence of outcome (MS relapse), death or December 31, 2016, whichever came first. MS relapse was defined as an occurrence of hospitalization with any diagnosis of MS as well as with treatment with pulse therapy (high dose steroids) or plasmapheresis/plasma exchange.

### Healthcare utilizations

Healthcare utilizations, including the number of outpatient visits, emergency room visits and hospitalizations within the first, second, and third years following the first diagnosis of MS, were evaluated. Only patients who had at least one record of utilization during the follow-up period were assessed in the analysis of healthcare utilization. For example, if a patient died before the year of follow-up or had no record of claims data during the year of follow-up, he or she would not be included in the analysis of utilization during that year. Healthcare utilizations were classified as MS-related, defined as visits or admissions with any diagnosis of MS, or non-MS-related.

### Statistical analysis

The number of incidence cases, age at diagnosis, and female-to-male sex ratio were calculated and reported annually for patients with MS between 2007 and 2016. Crude rates of incidence were presented annually as cases per 100,000 persons in the overall population of Taiwan and by sex and age group. Age-standardized incidence rates adjusted according to the WHO 2000–2025 standard population were also presented annually. Treatment patterns, including adherence to initial and subsequent therapy, were investigated and presented among total incidence cases, incidence cases during 2007–2010, and incidence cases during 2011–2014.

A Cox proportional hazard model was conducted to compare the risk of MS relapse between good and inadequate-to-poor adherence (reference group) in the early use of initial therapy. Model 1 was a crude estimate of the hazard ratio (HR) and 95% confidence interval (CI). Model 2 was adjusted for age and sex. Model 3 was adjusted for age, sex and a stepwise selection variable (a variable needs to be significant at the 0.25 level to be put in the model, while a variable in the model needs to be significant at the 0.15 level to remain in the model). Model 4 was adjusted for age, sex, the number of neurologist visits, depression, anxiety, hypertension, dyslipidemia, diabetes mellitus and the use of oral contraception. Model 5 was adjusted with all variables listed in Table 1.

**Table 1:** Baseline characteristics for incidence cases of multiple sclerosis.

	**Total**	
Patient number—N (%)	555	(100)
**Gender—N (%)**		
Male	142	(25.6)
Female	413	(74.4)
Pregnancy	12	(2.2)
**Age—N (%)**		
Mean (SD)	36.9	(12.1)
Median (Q1–Q3)	34.2	(26.8–45.2)
20–29	191	(34.4)
30–39	175	(31.5)
40 +	189	(34.1)
**No. of neurology outpatient visits—N (%)**		
Mean (SD)	0.5	(2.7)
Median (Q1–Q3)	0	(0–0)
0	513	(92.4)
1–5	25	(4.5)
6 +	17	(3.1)
**Comorbidities—N (%)**		
Depression	22	(4.0)
Anxiety	27	(4.9)
Migraine	12	(2.2)
Hypertension	60	(10.8)
Ischemic heart disease and heart failure	12	(2.2)
Cardiac dysrhythmia	5	(0.9)
Dyslipidemia	32	(5.8)
Diabetes mellitus	22	(4.9)
Obstructive lung disease (COPD, asthma)	11	(2.0)
Autoimmune disease	12	(2.2)
Malignancy	13	(2.3)
Traumatic brain injury	8	(1.4)
**Prior health behaviors—N (%)**		
Use of oral contraception	60	(10.8)

All data in this study were analyzed using SAS software, version 9.4 (SAS Institute, Cary, NC, USA).

## Results

### Study population

In our study, we identified 555 incidence MS cases (Fig. 1). To investigate treatment patterns and adherence, only MS cases identified between 2007 and 2014 (n = 486) were included to guarantee that the study population had at least 2 years of follow-up. For adherence analysis (n = 314), we excluded eight patients who died before the start of follow-up for MS relapse and 164 patients who did not receive initial therapy within 1 year after being newly diagnosed with MS. Among them, 78 were in the inadequate-to-poor adherence group, and 236 were in the good adherence group.

### Patient characteristics

As shown in Table 1, 74.4% of incidence cases of MS were females, with a mean age of 36.9 years. Due to the relatively younger age of our study population, the proportions of patients who had comorbidities were relatively low. For possible risk factors for MS, only 1.4% and 2.2% of our study population were diagnosed with traumatic brain injury and autoimmune diseases, respectively. Notably, 2.2% of patients experienced pregnancy within the baseline period.

### Incidence

During the 10-year study period (2007–2016), a slightly decreasing trend of incident cases (70 in 2007 to 24 in 2016) and crude incidence rate (0.41 in 2007 to 0.12 per 100,000 persons in 2016) can be observed (Table 2). For females, the incident cases and crude incidence were 32–42 cases and 0.34–0.44/100,000 persons during 2012–2015; for males, the incident cases and crude incidence were 8–16 cases and 0.09–0.18/100,000 persons during 2012–2015. The female-to-male sex ratios of incidence rates were between 1.72 and 5.05. The age-standardized incidence rates were similar to crude incidence rates.

**Table 2:** Incidence of multiple sclerosis in Taiwan.

	2007	2008	2009	2010	2011	2012	2013	2014	2015	2016
**Number of incidence cases—N (%)**										
Total	70 (100)	75 (100)	68 (100)	60 (100)	63 (100)	56 (100)	44 (100)	50 (100)	45 (100)	24 (100)
Age										
20–29	20 (28.6)	19 (25.3)	20 (29.4)	17 (28.3)	16 (25.4)	25 (44.6)	17 (38.6)	26 (52.0)	17 (37.8)	14 (58.3)
30–39	22 (31.4)	24 (32.0)	21 (30.9)	24 (40.0)	20 (31.7)	16 (28.6)	14 (31.8)	15 (30.0)	14 (31.1)	5 (20.8)
40 +	28 (40.0)	32 (42.7)	27 (39.7)	19 (31.7)	27 (42.9)	15 (26.8)	13 (29.5)	9 (18.0)	14 (31.1)	5 (20.8)
Gender										
Female	56 (80.0)	48 (64.0)	53 (77.9)	44 (73.3)	46 (73.0)	40 (71.4)	32 (72.7)	42 (84.0)	34 (75.6)	18 (75.0)
Male	14 (20.0)	27 (36.0)	15 (22.1)	16 (26.7)	17 (27.0)	16 (28.6)	12 (27.3)	8 (16.0)	11 (24.4)	6 (25.0)
**Crude incidence rate—per 100,000 person**										
Total	0.41	0.43	0.38	0.33	0.35	0.31	0.24	0.26	0.24	0.12
Age										
20–29	0.53	0.51	0.55	0.48	0.46	0.73	0.5	0.77	0.50	0.41
30–39	0.58	0.63	0.54	0.62	0.50	0.40	0.34	0.36	0.34	0.12
40 +	0.29	0.32	0.26	0.18	0.25	0.14	0.12	0.08	0.12	0.04
Gender										
Female	0.64	0.54	0.59	0.48	0.5	0.43	0.34	0.44	0.35	0.18
Male	0.16	0.31	0.17	0.18	0.19	0.18	0.13	0.09	0.12	0.06
Sex ratio										
Female:Male	3.88	1.72	3.41	2.65	2.60	2.40	2.57	5.05	2.97	2.88
**Age-standardized incidence rate** ^a^**—per 100,000 person**										
Total	0.41	0.44	0.40	0.35	0.36	0.34	0.26	0.31	0.26	0.15

^a^According to WHO 2000–2025 standard population.

### Treatment patterns

There were 81.5% of incidence cases who received initial therapy for MS, and the proportion of those who had received initial treatment was 86.9% among incidence cases during 2011–2014 (Table 3). Most patients (64.9%) were administered IFN β-1a as their initial treatment; compared with 57.3% during 2007–2010, a greater proportion of incidence cases (73.5%) received IFN β-1a as the initial treatment during 2011–2014. IFN β-1b was the second most commonly used treatment, but the result shows a decreasing trend of IFN β-1b being prescribed as initial treatment during the study period. The median time between the diagnosis of MS and initial treatment was 77 days. The time interval between diagnosis and the initiation of initial treatment was shortened during 2011–2014 compared with 2007–2010.

**Table 3:** Treatment patterns of incidence cases of multiple sclerosis.

	**Total**		**2007–2010**		**2011–2014**	
**Patient number—N (%)**	486	(100)	273	(100)	213	(100)
Patients receiving initial therapy	396	(81.5)	211	(77.3)	185	(86.9)
Patients receiving subsequent therapy	161	(33.1)	100	(36.6)	61	(28.6)
**Initial therapy**						
Type of initial therapy—N (%)						
Overall	396	(100)	211	(100)	185	(100)
Interferon β-1a	257	(64.9)	121	(57.3)	136	(73.5)
Interferon β-1b	86	(21.7)	57	(27.0)	29	(15.7)
Others^a^	53	(13.4)	33	(15.6)	20	(10.8)
Time from first diagnosis to initial therapy (days)						
Mean (SD)	269.6	(481.8)	344.9	(601.1)	183.8	(267.1)
Median (Q1–Q3)	77	(35–280)	84	(33–333)	70	(35–188)
**Subsequent therapy**						
Type of subsequent therapy—N (%)						
Overall	161	(100)	100	(100)	61	(100)
Fingolimod	102	(63.4)	58	(58.0)	44	(72.1)
Interferon β-1a	27	(16.8)	20	(20.0)	7	(11.5)
Others^b^	32	(19.9)	22	(22.0)	10	(16.4)
Time from initial therapy to subsequent therapy (days)						
Mean (SD)	1144.2	(824.8)	1413.9	(854.9)	702.0	(536.5)
Median (Q1-Q3)	1065	(441–1708)	1403	(702–2051)	528	(282–1183)

^a^Glatiramer acetate, fingolimod and azathioprine.

^b^Natalizumab, glatiramer acetate, interferon β-1b, azathioprine, mycophenolate mofetil, mitoxantrone and rituximab.

Approximately 33.1% of incidence cases had received subsequent treatment for MS (Table 3). Among those who had been prescribed subsequent treatment, 63.4% of them received fingolimod. There was an apparent increasing trend regarding the proportion of fingolimod as a subsequent treatment among incidence cases during 2011–2014 compared with 2007–2010. The median time interval between initial therapy and subsequent therapy was 1065 days. Notably, the time interval was significantly shortened during 2011–2014 compared with 2007–2010.

### Adherence

Overall, 73.5% of patients had good adherence (MPR ≥ 0.8) to their initial therapy at 12 months following the initial therapy (Table 4). At 24 months following the initiation of the initial therapy, an overall 10% drop in the good adherence group was noticed. Among incidence cases who had ever been prescribed subsequent therapy, 66.5% of them had good adherence (MPR ≥ 0.8) to subsequent therapy at 12 months following the subsequent therapy. At 24 months following the initiation of the subsequent therapy, an overall 10% drop in the good adherence group was also observed.

**Table 4:** Adherence of initial therapy and subsequent therapy.

	Total		2007–2010		2011–2014	
**Initial therapy**						
Patient number—N (%)	396	(100)	211	(100)	185	(100)
12 months following the initial therapy—N (%)						
Good adherence (MPR ≥ 0.8)	291	(73.5)	152	(72)	139	(75.1)
Inadequate adherence (0.8 > MPR ≥ 0.4)	46	(11.6)	25	(11.8)	21	(11.4)
Poor adherence (MPR < 0.4)	59	(14.9)	34	(16.1)	25	(13.5)
MPR						
Mean (SD)	0.92	(0.72)	0.88	(0.37)	0.97	(0.98)
Median (Q1-Q3)	1.01	(0.75–1.08)	1.01	(0.72–1.08)	0.99	(0.81–1.09)
24 months following the initial therapy—N (%)						
Good adherence (MPR ≥ 0.8)	247	(62.4)	136	(64.5)	111	(60.0)
Inadequate adherence (0.8 > MPR ≥ 0.4)	61	(15.4)	28	(13.3)	33	(17.8)
Poor adherence (MPR < 0.4)	88	(22.2)	47	(22.3)	41	(22.2)
MPR						
Mean (SD)	0.80	(0.54)	0.78	(0.39)	0.81	(0.67)
Median (Q1-Q3)	0.94	(0.47–1.04)	0.94	(0.51–1.03)	0.93	(0.44–1.04)
**Subsequent therapy**						
Patient number—N (%)	161	(100)	100	(100)	61	(100)
12 months following the subsequent therapy—N (%)						
Good adherence (MPR ≥ 0.8)	107	(66.5)	67	(67.0)	40	(65.6)
Inadequate adherence (0.8 > MPR ≥ 0.4)	17	(10.6)	12	(12.0)	5	(8.2)
Poor adherence (MPR < 0.4)	37	(23.0)	21	(21.0)	16	(26.2)
MPR						
Mean (SD)	0.83	(0.44)	0.82	(0.37)	0.86	(0.53)
Median (Q1-Q3)	0.99	(0.54–1.08)	0.99	(0.57–1.05)	1.01	(0.39–1.09)
24 months following the subsequent therapy—N (%)						
Good adherence (MPR ≥ 0.8)	86	(53.4)	60	(60.0)	26	(42.6)
Inadequate adherence (0.8 > MPR ≥ 0.4)	31	(19.3)	13	(13.0)	18	(29.5)
Poor adherence (MPR < 0.4)	44	(27.3)	27	(27.0)	17	(27.9)
MPR						
Mean (SD)	0.71	(0.42)	0.75	(0.43)	0.66	(0.40)
Median (Q1–Q3)	0.86	(0.34–1.02)	0.94	(0.35–1.05)	0.71	(0.23–1.00)

### Adherence analysis

We followed up 236 incidence cases in the good adherence group and 78 incidence cases in the inadequate-to-poor adherence group of early uses of initial therapy to compare their risk of MS relapses. Patient characteristics of the two groups are presented in Supplement 1. The crude HR of the good adherence group compared with the inadequate-to-poor group was 0.76 (95% CI 0.49–1.20) (Table 5). Model 2 adjusted for age and sex, with an HR of 0.74 (95% CI 0.47–1.16), while model 3, model 4 and model 5 had similar estimates of HRs, 0.77 (95% CI 0.49–1.21), 0.76 (95% CI 0.48–1.21) and 0.77 (95% CI 0.48–1.22), respectively, when adjusted for chosen variables. Although all 5 models showed no statistical significance, the good adherence group tended to have a lower risk of MS relapse in terms of the point estimates.

**Table 5:** Adherence analysis: association between adherence and occurrence of MS relapse.

	Model 1^a^			Model 2^b^			Model 3^c^			Model 4^d^			Model 5^e^		
	HR	95% CI	p-value	HR	95% CI	p-value	HR	95% CI	p-value	HR	95% CI	p-value	HR	95% CI	p-value
**Adherence**															
Inadequate-to-poor	Ref			Ref			Ref			Ref			Ref		
Good	0.76	(0.49–1.20)	0.24	0.74	(0.47–1.16)	0.19	0.77	(0.49–1.21)	0.26	0.76	(0.48–1.21)	0.25	0.77	(0.48–1.22)	0.26

*Ref* reference, *HR* hazard ratio, *CI* confidence interval.

^a^Model 1: crude.

^b^Model 2: adjusted with age and sex.

^c^Model 3: adjusted with age, sex and stepwise selection variable (use of oral contraception).

^d^Model 4: adjusted with age, sex, number of neurologist visits, depression, anxiety, hypertension, dyslipidemia, diabetes mellitus, use of oral contraception.

^e^Model 5: adjusted with full variables.

### Healthcare utilizations

Results of healthcare utilizations are presented in Supplement 2. Among incidence cases, the number of MS-related and non-MS-related visits to outpatient departments was significantly higher than visits to ERs and hospitalizations during the first, second, and third years following the first MS diagnosis. The proportion of patients who had MS-related outpatient visits declined (from 96.8 to 89.4%) over the 3-year follow-up period. The proportion of patients who had MS-related ER visits and hospitalizations was highest during the first year. On the other hand, the number of non-MS-related outpatient visits, ER visits and hospitalizations remained stable over the 3-year follow-up period.

## Discussion

To the best of our knowledge, this is the first population-based epidemiological study conducted in Asia focusing on the incidence of MS. Epidemiological studies on MS have been thoroughly conducted and kept updated during the past few decades in North America and Europe^1–4,6–10^, and some previous studies carried out in Asia also looked at the incidence of MS over the past couple of years^13–19^. However, our study provides more recent data on the epidemiology of MS and treatment patterns as well as healthcare utilizations of MS patients, which have seldom been investigated in existing studies.

Our study found that the age-standardized incidence rate ranged from 0.15 to 0.44 per 100,000 persons during 2007–2016, which was much lower than that in Italy (0.7–9.2 per 100,000 persons), the British Isles (7.2–12.2 per 100,000 persons), the Nordic region (1.28–11.6 per 100,000 persons), Belgium and France (4.3–10.8 per 100,000 persons), other central European countries (6–7.7 per 100,000 persons)^9^, and the US (34.8–46.3 per 100,000 persons)^33^. However, the incidence of MS in Taiwan was relatively comparable to that in other Southeast European countries (0.32–1.6 per 100,000 persons)^9^. Compared to other Asian countries, the incidence in Taiwan was lower than that in Japan (0.06–0.78 per 100,000 persons)^13^ but higher than that in Korea (0.1 per 100,000 persons)^14^. The variations in latitudes might explain the differences in MS incidence among central and northern European countries, the US, and Taiwan^34^. However, other environmental factors, together with genetic factors, such as vitamin D, Epstein-Barr virus and cigarette smoking, might also be possible explanations^35^. The lower incidence of cases since 2010 in our study might result from the updated McDonald criteria in 2010, in which patients need to fulfill stricter criteria to be diagnosed as having MS^36^. In the current study, the female-to-male sex ratios of crude incidence rates ranged from 1.72 to 5.05 and were mainly between 2 and 3, which seemed to be similar to the finding in Europe, in which the female-to-male sex ratio of incidence was as high as 3^9^.

In terms of the age group of incidence cases, we found an increasing proportion of patients diagnosed with MS during their 20 s (from 28.6% in 2007 to 58.3% in 2016), indicating a trend of diagnosis at an early age. Adopting the updated 2010 McDonald Criteria in clinical practice may be a reasonable explanation for this trend. However, more studies may be warranted to figure this out^36^. On the other hand, the time interval between diagnosis and initial therapy had shortened among incidence cases during 2011–2014 compared with 2007–2010 in Taiwan. Evidence has shown that early diagnosis and early treatment may delay the negative impact of MS, including axonal damage, brain atrophy, and irreversible neurological disability^37,38^. The trends of both diagnosis at an early age and early initiation of therapy found in our study may give us a picture that MS management has improved in Taiwan during recent years, which may further improve the prognosis of MS patients.

Patients received mainly fingolimod as subsequent therapy in our study. This may explain why the time interval between initial therapy and subsequent therapy decreased intensively among incidence cases during 2011–2014 compared with 2007–2010. Fingolimod, which was approved and enrolled in the reimbursement scheme of the National Health Insurance program in 2012 as the first oral agent for the treatment of MS in Taiwan, is a second-line treatment that is used when a first-line treatment of IFN β or glatiramer acetate fails to reduce MS relapses. For incidence cases during 2007–2010, even though their first-line therapy failed, they were not able to receive fingolimod until 2012, while incidence cases during 2011–2014 may have received fingolimod once their first-line treatment failed. Compared with a study investigating treatment patterns in MS in the US during 2001–2010, similar patterns were observed in most MS patients who were users of IFN β-1a and IFN β-1b; however, they tended to transfer to the use of IFN, GA or natalizumab rather than to fingolimod^26^.

In a study conducted in Germany, 32.3% of patients with MS who initiated DMTs during 2002–2006 had an MPR ≥ 0.8 during the observation period of 24 months^28^, which was lower than what we observed following 24 months in the current study, regardless of initial therapy or subsequent therapy. On the other hand, among patients in Canada who received first-line therapy (including IFN β-1b, IFN β-1a and GA) during the study period of 1996–2014, 76.4% of patients had a proportion of days covered^29^ ≥ 0.8 at an observation time of one year, which was comparable to adherence following 12 months of initial therapy in the current study.

Although the estimates were not statistically significant, probably due to the relatively small sample size, the results of adherence analysis showed a tendency of a lower risk of MS relapse in patients with good adherence to initial therapy than in patients with inadequate-to-poor adherence. This may point out that not only early initiation of therapy but also good adherence to therapy is crucial for the management of MS. Previous studies have shown that better adherence to DMTs is significantly related to lower rates of MS relapse, hospitalizations and medical costs^22,23,25^.

For healthcare utilizations, the proportion of patients who had MS-related hospitalizations during the first year following the diagnosis of MS was much higher than that of patients who had MS-related hospitalizations during the second and third years; this finding may give us a message that patients first diagnosed with MS need more intense treatments or follow-ups to prevent events requiring hospitalizations.

The limitations of this study should be addressed. First, as many studies using claims data, data not included in claims database such as laboratory data, was not captured. In this way, we are not able to evaluate the severity of MS. In addition, we could not guarantee medications being filled are taken eventually. Another inherent limitation of the claims data is that it should be more cautious when interpreting incidence at the beginning and end of the study period. For those who were identified as incident cases at the beginning of our study period (e.g. 2007–2008), they were with relatively shorter wash-out period and were misclassified as incident cases. On the other hand, for patients at the end of our study period (e.g. 2007–2008), there were no enough follow-up period for potential cases to be identified as MS cases as the diagnosis of MS requires some time. Therefore, incidence from 2009 to 2014 (0.38–0.26 per 100,000 person) would be considered more solid for the interpretation. Third, people aged 20 and above are legally considered as adults in Taiwan. Due to ethical consideration, data access to patients aged under 20, which are children and teenagers, is considered as vulnerable populations and required a strict application process. Therefore, our study only included adult MS patients. Fourth, we excluded patients not receiving any MS treatment within 12 months after diagnosis in the adherence analysis. Although this is to mitigate potential confounding by indication bias due to comparisons of the adherence between those who received treatment and those who did not, this may overestimate the adherence of these MS patients. Finally, healthcare utilizations regarding MS-related visits or hospitalizations might be overestimated, as we relied on diagnosis codes to distinguish whether the visits were MS-related or not.

Nevertheless, there are merits of our study. Using the population-based data source, our results could provide a nationwide estimate for MS, which could serve as a good reference for health policy. Moreover, we used patients in the Registry for Catastrophic Illness to confirm the diagnosis of MS, and we applied more stringent inclusion/exclusion criteria to exclude patients with NMOSD, establishing a more accurate estimate of the MS study population. We also provide more recent data on the epidemiology of MS and treatment patterns as well as healthcare utilization of MS patients, which have seldom been investigated in existing studies. Thus, our study thus fills the knowledge gap regarding the whole picture of MS, particularly in Asia.

## Conclusions

The current study is the first population-based study conducted in Asia to provide valuable data of the epidemiology and treatment patterns of MS. These “real-world data” could serve as a good reference for improving MS management and fostering related health policy.

## Supplementary Information

Supplementary Information
