# Supplementary material for: Epidemiology, treatment patterns and healthcare utilizations in multiple sclerosis in Taiwan
Source: Sci Rep. 2021 Apr 8;11:7727. doi: 10.1038/s41598-021-86347-3 (PMC8032718; doi:10.1038/s41598-021-86347-3)
Supplement: Supplementary file 1 — Supplementary Information [file 41598_2021_86347_MOESM1_ESM.pdf]

Epidemiology, treatment patterns and healthcare utilizations in multiple sclerosis in Taiwan

Chia-Yun Hsu[1#], Long-Sun Ro[2#], Li-Ju Chen[1], Chun-Wei Chang[2], Kuo-Hsuan Chang[2], I-Hsuan Wu[3], Amy Lin[3], Fei-Yuan Hsiao[4,5,6*]

[1] Health Data Research Center, National Taiwan University, Taipei, Taiwan

[2] Department of Neurology, Chang Gung Memorial Hospital, Taipei, Taiwan

[3] Merck Ltd., Taipei, Taiwan

[4] Graduate Institute of Clinical Pharmacy, College of Medicine, National Taiwan University, Taipei, Taiwan

[5] School of Pharmacy, National Taiwan University, Taipei, Taiwan

[6] Department of Pharmacy, National Taiwan University Hospital, Taipei, Taiwan

[#] These authors contributed equally.

*Corresponding author:

Fei-Yuan Hsiao, Ph.D.

Graduate Institute of Clinical Pharmacy,

College of Medicine, National Taiwan University

Room 220, 33, Linsen S. Rd, Taipei, Taiwan 10050

TEL: +886-2-33668787

FAX: +886-2-33668780

Email: fyshsiao@ntu.edu.tw

Supplement 1. Patient characteristics for adherence analysis

	Inadequate to poor adherence (MPR<0.8)		Good adherence (MPR≥0.8)	
**Patient number – N (%)**	78	(100)	236	(100)
**Gender – N (%)**				
Male	20	(25.6)	64	(27.1)
Female	58	(74.3)	172	(72.9)
Pregnancy				
**Age – N (%)**				
Mean (SD)	37.27	(11.8)	35.62	(11.2)
Median (Q1-Q3)	36.18	(27.6-43.9)	33.17	(26.4-43.4)
20-29	25	(32.1)	87	(36.9)
30-39	30	(38.5)	74	(31.4)
40+	23	(29.5)	75	(31.8)
**Neurology OPD visits – N (%)**				
Mean (SD)	0.8	(3.2)	0.62	(2.8)
Median (Q1-Q3)	0	(0-0)	0	(0-0)
0	70	(89.7)	217	(92.9)
1-5	4	(5.1)	10	(4.2)
6+	4	(5.1)	9	(3.8)
**Comorbidities – N (%)**				
Depression	4	(5.1)	11	(4.7)
Hypertension	7	(9.0)	23	(9.8)
Dyslipidemia	5	(6.4)	12	(5.1)
Diabetes mellitus	4	(5.1)	7	(3.0)
Obstructive lung disease	3	(3.9)	3	(1.3)
Autoimmune disease	3	(3.9)	4	(1.7)
Anxiety [a]	-	-	-	-
Migraine [a]	-	-	-	-
Ischemic heart disease and heart failure [a]	-	-	-	-
Cardiac dysrhythmia [a]	-	-	-	-
Malignancy [a]	-	-	-	-
Traumatic brain injury [a]	-	-	-	-
**Priority health behaviors – N (%)**				
Use of oral contraception	12	(15.4)	18	(7.6)

[a] Patient number less than 3 and related data were not able to be shown in the table due to the privacy policy of data analysis

Supplement 2. Healthcare utilization of incidence cases of MS following first diagnosis

	Year 1	Year 2	Year 3
**All patient number – N (%)**	555 (100)	517 (100)	463 (100)
**Outpatient visits**			
Patient number of outpatient visit – N (%)	552 (99.5)	517 (100)	463 (100)
MS-related visit			
**Number of visit**			
Patient number of MS-related visit – N (%)	537 (96.8)	469 (90.7)	414 (89.4)
Mean [a] (SD)	12.7 (9.5)	10.4 (8.9)	9.7 (7.8)
Median (Q1-Q3)	11 (7-17)	8 (5-14)	7 (5-13)
Non-MS-related visit			
**Number of visit**			
Patient number of non-MS-related visit – N (%)	532 (95.9)	501 (96.9)	438 (94.6)
Mean [b] (SD)	15 (14.1)	14.1 (14.2)	14.1 (14.2)
Median (Q1-Q3)	10.5 (5-19.5)	10 (5-19)	10 (5-19)
**ER visits**			
Patient number of ER visit – N (%)	232 (41.8)	142 (27.5)	132 (28.5)
MS-related visit			
**Number of visit**			
Patient number of MS-related visit – N (%)	123 (22.2)	79 (15.3)	72 (15.6)
Mean [a] (SD)	1.7 (1.5)	2.5 (4.3)	1.8 (1.7)
Median (Q1-Q3)	1 (1-2)	1 (1-3)	1 (1-2)
Non-MS-related visit			
**Number of visit**			
Patient number of non-MS-related visit – N (%)	156 (28.1)	88 (17.0)	73 (15.8)
Mean [b] (SD)	1.5 (1.1)	1.7 (2.1)	1.4 (0.8)
Median (Q1-Q3)	1 (1-2)	1 (1-2)	1 (1-2)
**Hospitalization**			
Patient number of hospitalization – N (%)	440 (79.3)	147 (28.4)	112 (24.2)
MS-related admission			
**Number of admission**			
Patient number of MS-related admission – N (%)	432 (77.8)	129 (25.0)	96 (20.7)
Mean [a] (SD)	1.6 (1.2)	1.7 (1.1)	1.8 (1.8)
Median (Q1-Q3)	1 (1-2)	1 (1-2)	1 (1-2)
**Length of stay**			
Mean [c] (SD)	12 (13.4)	11.2 (26.1)	24 (177.2)
Median (Q1-Q3)	7 (5-14)	7 (4-10)	6 (4-14)
Non-MS-related admission			
**Number of admission**			
Patient number of non-MS-related admission – N (%)	41 (7.4)	32 (6.2)	23 (5.0)

	Year 1	Year 2	Year 3
Mean [b] (SD)	1.5 (1)	1.5 (1)	2.1 (1.9)
Median (Q1-Q3)	1 (1-2)	1 (1-1.5)	1 (1-2)
**Length of stay**			
Mean [c] (SD)	37.8 (154.4)	13.9 (37)	25.8 (106.9)
Median (Q1-Q3)	8 (3-27)	4 (2-8)	6 (2-12)

[a] Mean number per patient having MS-related visit/admission

[b] Mean number per patient having non-MS-related visit/admission

[c] Mean length of stay per admission

Supplement 3. Cost of healthcare utilization of incidence cases of MS following first diagnosis

	Year 1	Year 2	Year 3
**All patient number – N (%)**	555 (100)	517 (100)	463 (100)
**Outpatient visits**			
Patient number of outpatient visit – N (%)	552 (99.5)	517 (100)	463 (100)
MS-related visit			
**Cost of visit** [a]			
Patient number of MS-related visit – N (%)	537 (96.8)	469 (90.7)	414 (89.4)
Mean (SD)	238,394 (195,540)	286,604 (219,671)	290,016 (227,983)
Median (Q1-Q3)	240,624 (27,128-415,199)	326,693 (34,419-465,288)	311,640 (43,146-464,728)
**Cost of DMD medication** [a]			
Mean (SD)	212,858 (190,905)	264,565 (216,863)	267,213 (225,007)
Median (Q1-Q3)	222,017 (0-375,240)	300,192 (0-450,288)	277,620 (0-450,288)
Non-MS-related visit			
**Cost of visit** [b]			
Patient number of non-MS-related visit – N (%)	532 (95.9)	501 (96.9)	438 (94.6)
Mean (SD)	15,354 (23,250)	12,584 (25,548)	11,784 (19,234)
Median (Q1-Q3)	8,809 (3,785-18,440)	6,638 (2,788-13,674)	6,285 (2,930-13,788)
**ER visits**			
Patient number of ER visit – N (%)	232 (41.8)	142 (27.5)	132 (28.5)
MS-related visit			
**Cost of visit** [a]			
Patient number of MS-related visit – N (%)	123 (22.2)	79 (15.3)	72 (15.6)
Mean (SD)	8,659 (9,584)	11,140 (14,963)	8,511 (9,525)
Median (Q1-Q3)	4,487 (2,008-12,171)	3,956 (1,773-15,409)	4,712 (1,662-13,372)
**Cost of DMD medication** [a]			

	Year 1	Year 2	Year 3
Mean (SD)	26 (283)	105 (674)	71 (434)
Median (Q1-Q3)	0 (0-0)	0 (0-0)	0 (0-0)
Non-MS-related visit			
**Cost of visit [b]**			
Patient number of non-MS-related visit – N (%)	156 (28.1)	88 (17.0)	73 (15.8)
Mean (SD)	5,627 (5,839)	4,739 (7,719)	3,676 (3,768)
Median (Q1-Q3)	3,094 (1,400-7,790)	2,045 (1,138-5,162)	2,285 (1,245-3,717)
**Hospitalization admissions**			
Patient number of hospitalization – N (%)	440 (79.3)	147 (28.4)	112 (24.2)
MS-related admission			
**Cost of admission [a]**			
Patient number of MS-related admission – N (%)	432 (77.8)	129 (25.0)	96 (20.7)
Mean (SD)	107,572 (219,101)	93,279 (164,042)	192,020 (974,756)
Median (Q1-Q3)	52,358 (33,337-94,148)	50,078 (30,724-85,082)	45,199 (26,173-105,908)
**Cost of DMD medication [a]**			
Mean (SD)	4,570 (17,755)	9,052 (24,116)	11,561 (42,297)
Median (Q1-Q3)	0 (0-0)	0 (0-9,381)	0 (0-2,049)
Non-MS-related admission			
**Cost of admission [b]**			
Patient number of non-MS-related admission – N (%)	41 (7.4)	32 (6.2)	23 (5.0)
Mean (SD)	282,735 (809,879)	89,559 (152,175)	171,624 (351,723)
Median (Q1-Q3)	42,317 (19,645-160,202)	31,786 (15,789-82,786)	49,982 (25,625-107,166)

[a] Cost per patient having MS-related visit/admission

[b] Cost per patient having non-MS-related visit/admission
